# Marginal bone loss around axial and straight implants supported with prefabricated SFI-Bar with mandibular overdentures

**DOI:** 10.6026/97320630017289

**Published:** 2021-01-31

**Authors:** Prema Balehonnur, Vaibhav Nandakumar Awinashe, Anuj Singh Parihar, Doddy Lokanathan Balaji, Anuraj Singh Kochhar, Rajat Mehndiratta

**Affiliations:** 1 Department of Prosthodontics, Government dental college and research Institute, Bangalore, Karnataka, India; 2 Department of Prosthodontics, College of dentistry in Ar Rass, Qassim University, Kingdom of Saudi Arabia; 3 Department of Periodontology and Oral Implantology, Peoples Dental Academy Bhopal, MP, India; 4 Department of Prosthodontics, Priyadarshini Dental College and Hospital,Thiruvallur, 631203, India

**Keywords:** Axial, Implants, mandibular overdentures

## Abstract

To assess the role of prefabricated SFI-Bar in peri-implant bone loss around immediately axially loaded and straight implants. This study comprised of 40 complete denture wearer patients who received two axially parallel implants connected by SFI-Bars in group I and two 15° mesially tilted implants connected by SFI-Bars in group II. Peri- implant bone loss (PiBL) was measured at 1 year, 2 years and 3 years. The mean PiBL at 1 year in group I was 0.21 mm and I group II was 0.22, at 2 years in group I was 0.26 mm and in group II was 0.23 mm and at 3 years, in group I was 0.29 mm and in group II was 0.34 mm. The difference was significant at 3 years (P< 0.05). The mean mesial PIBL at 1 year in group I was 0.18 mm, in group II was 0.20 mm, at 2 years in group I was 0.19 mm and in group II was 0.07 mm and at 3 years, in group I was 0.25 mm and in group II was 0.29 mm. The difference found to be significant in each time duration in both groups (P< 0.05).The mean distal PIBL at 1 year in group I was 0.23 mm, in group II was 0.22 mm, at 2 years in group I was 0.33 mm and in group II was 0.39 mm and at 3 years, in group I was 0.34 mm and in group II was 0.39 mm. The difference found to be significant at 2 and 3 years in both groups (P< 0.05). Authors found that mandibular overdentures retained with Prefabricated SFI-Bar with axial and straight inserted implants may be useful in patients with reduced bone height.

## Background

With the overall increase in life expectancy of Indian population, the number of edentulous patients had rise significantly. Dental implants in partially and completely edentulous patients have become treatment of choice nowadays [1]. Implant dentistry has proved beneficial in such patients. Implant overdentures is considered to be alternative method in patients with severe residual alveolar bone loss. Improved patient's acceptance and successful treatment response had obtained with two implants inserted in mandible and subsequently placing overdenture [2]. Immediate loaded implants supporting overdentures are widely used these days, thus reduced the overall treatment duration, cost and limited complications. Splinting bars, double crowns, ball attachment and stud type such as locators and magnets are different attachment systems for implant overdentures [3]. In old patients with severe residual alveolar bone loss, bar attachments and double crowns proved to be advantageous. Bar attachments in implant supported overdentures offer less bending movements by permitting rotation of the denture base around the supporting implants [4]. Certain oral conditions such as excessive bleeding from plant site, mucosal swelling etc. pose difficulties in obtaining impressions. Subsequently, there may be altered fabrication of laboratory processed conventional bars. Tilted implants may be used in conditions where there is compromised ridge contour [5]. Stress free implant bar (SFI Bar) has the advantages of both bars and study attachment systems. It is a prefabricated attachment system that containing two or more implants attached to each other without soldering or laser welded joints [6]. It is a chair side procedure that does not require impression. SFI bar adaptor allows 30° for the tilted implants and permits fabrication of a passive fit bar and clip system. This system minimizes the force transfer to the implants and results in less marginal bone loss [7]. Therefore, it is of interest to assess the role of prefabricated SFI-Bar in peri-implant bone loss around immediately axially loaded and straight implants.

## Methodology

This study comprised of 40 complete denture wearer patients of both genders who were recruited after obtaining their written consent. Patients with not less than 1.5 cm of bone height in anterior mandible, without TMJ disorders and patients with not less than 1.4 cm vertical space from alveolar crest at the proposed implant sites to the incisal edge of the artificial teeth and patients with good quality bone. Ethical approval was obtained from ethical clearance committee. The restorative space ie the distance from the undersurface of mandibular dentures to their incisal edges was calculated with Boley gauge. A mucosally supported stereolithographic surgical guide for flapless implant placement was created with CBCT virtually. Each implant was inserted axially at least 5 mm away from the anterior wall of the mental nerve loops bilaterally or tilted mesially at 15° from the vertical axis. Patients were classified into groups. Group I comprised of 20 patients (males- 12, females- 8) who received two axially parallel implants connected by SFI Bars and group II comprised of 20 patients (males- 9, females- 11) received two 15° mesially tilted implants connected by SFI Bars. Following all standardized surgical procedures, 2 implant fixtures were inserted on both left and right side in the canine regions (MIS implant, USA). The implant insertion speed of 30rpm was used form insertion and 80 Ncm torque was applied with torque wrench. With resonance frequency analysis, the implant stability was checked. SFI- bars hold both implants in canine region bilaterally and mandibular denture was loaded immediately. Patients were discharge after prescribing antibiotics, cap. Amoxicillin 500 mg thrice a days and anti- inflammatory diclofenac sodium 50 mg twice a day for 5 days. Chlorhexidine mouth was 0.2% once a day was also recommended. Patients were recalled regularly and peri- implant bone loss (PiBL) was measured at after 1 year till 3 years. Results were subjected to statistical analysis.

## Results:

It is of interest to assess role of prefabricated SFI-Bar in peri-implant bone loss around immediately axially loaded and straight implants. This study comprised of 40 complete denture wearer patients who received two axially parallel implants connected by SFI-Bars in group I and two 15° mesially tilted implants connected by SFI-Bars in group II. Peri- implant bone loss (PiBL) was measured at 1 year, 2 years and 3 years. The mean PiBL at 1 year in group I was 0.21 mm and I group II was 0.22, at 2 years in group I was 0.26 mm and in group II was 0.23 mm and at 3 years, in group I was 0.29 mm and in group II was 0.34 mm. The difference was significant at 3 years (P< 0.05) (Table 1 - see PDF).

The mean mesial PIBL at 1 year in group I was 0.18 mm, in group II was 0.20 mm, at 2 years in group I was 0.19 mm and in group II was 0.07 mm and at 3 years, in group I was 0.25 mm and in group II was 0.29 mm. The difference found to be significant in each time duration in both groups (P< 0.05) (Graph-1). The mean distal PIBL at 1 year in group I was 0.23 mm, in group II was 0.22 mm, at 2 years in group I was 0.33 mm and in group II was 0.39 mm and at 3 years, in group I was 0.34 mm and in group II was 0.39 mm. The difference found to be significant at 2 and 3 years in both groups (P< 0.05) (Grraph-2). Authors found that mandibular over dentures retained with Prefabricated SFI-Bar with axial and straight inserted implants may be useful in patients with reduced bone height.

## Discussion:

SFI Bar design allows better cleaning, as there is minimal accumulation of dental plaque. This system allows tilting of dental implants, which is highly advantageous especially in patients with compromised alveolar ridge height [8]. Moreover, this system permits better transfer of stress along implants thus ensuring survival rate. Thus giving intentional angulations to implants may be helpful. However, delayed loading of implants with the invasive grafting procedures can also be considered alternate [9]. It is evident that initial primary stability of implant retained overdentures is greatly affected by the transmitted occlusal forces along it through splinting effect. This mechanism ensures long term success rate of implants [10]. The present study was conducted to assess the role of prefabricated SFI Bar in peri implant bone loss around immediately loaded bilateral axially implants and mesially tiled implants.

This study comprised of 40 patients distributed in 2 groups of 20 each. In group I, two axially parallel implants connected by SFI Bars and group II, two 15° mesially tilted implants connected by SFI Bars were used. Abdel et al.[11] studied 30 patients who got either axial implants or mesially tilted implants connected by SFI Bars for retaining mandibular overdentures in canine region bilaterally. Patients were recalled to assess bone loss. Both groups showed insignificant PiBL at 12 and 24 months, while at 36 months, patients in Group TB showed significantly higher marginal PiBL than that with Group AB. We found that mean PiBL at 1 year in group I was 0.21 mm and I group II was 0.22, at 2 years in group I was 0.26 mm and in group II was 0.23 mm and at 3 years, in group I was 0.29 mm and in group II was 0.34 mm. The difference was significant at 3 years (P< 0.05). We observed that maximum bone loss occurred in 1 years and our results are in consistence with Sannino et al. [12].

Lehmann et al. [13] assessed plaque index, Sulcus Bleeding Index, probing pocket depth (PPD), and peri-implant bone loss in patients who received TiOblast implants supported overdentures retained by prefabricated bars (group A) with or without extensions (group B) and cast bars (Group C). Results showed PI, SBI, PPD, and pathologic bone loss were least common in the group A, followed by group B, and group C. It was found that plaque accumulation and pathologic bone loss values were higher with implants that supported mandibular bar-retained overdentures than with those supporting maxillary bar-retained overdentures (P > 0.05).

We observed that mean mesial PIBL in group I was 0.18 mm, 0.19 mm and 0.25 mm at 1 year, 2 years and 3 years respectively. In group II was 0.20 mm, 0.07 mm and 0.29 mm at 1 year, 2 years and 3 years respectively. The difference found to be significant in both groups (P< 0.05). Similarly, mean distal PIBL at 1 year, 2 years and 3 years was as (group I- 0.23 mm, group II- 0.22 mm, (group I- 0.33 mm, group II- 0.39 mm) and (group I- 0.34 mm and group II- 0.39 mm respectively. The difference found to be significant at 2 and 3 years in both groups (P< 0.05).

Monje A et al. [14] in their study evaluated results found in 8 studies with 1,015 implants. Implants were either tilted or straight depending upon the requirement. Study results showed that there was more marginal bone loss with tiled implants in comparison to straight implants, however it was non- significant. Tözüm et al. [15] studied 17 completely edentulous patients who received either two ball attachment mandibular overdentures and early and delayed-loaded dental implants were inserted. There was a negative correlation between RFA measurements and marginal bone level, whereas some correlations also existed between RFA and PISF volume. The pattern of loading found the relationship between RFA measurements and marginal bone level. Wang et al. estimated the effect of the implant lengths and sleeve lengths on accurateness of static computer-assisted implant surgery (sCAIS). 55 implants were positioned under the guidance of sCAIS. They found no significant variance in implant vertical aberration between dissimilar sleeve height groups (1-10 mm). They concluded that, length of the sleeves has substantial effect on the correctness of the surgical guide [16]. Singh et al. conducted a study to compare 3D miniplate system with 2D plates in mandibular angle fractures treatment. They concluded that 3D miniplate system is consistent and efficient for mandibular angle fractures as related with traditional 2D miniplates [17]. Todescan et al. observed the relationships and dimensions of the peri-implant tissues adjacent to osseointegrated 2-stage implants positioned at various depths in bone. In 4 mongrel dogs, 24 implants were positioned in the mandible. Histologic clarifications presented a mucosal obstruction consisting of keratinized oral epithelium. They concluded that, there was a clear adaptation of the connective tissue and epithelium with deeper implants [18]. The shortcoming of present study is small sample size. The long follow up was not done. The effect of surrounding soft tissues on implant outcome was not taken into consideration.

## Conclusion

Data shows that mandibular overdentures retained with Prefabricated SFI-Bar with axial and straight inserted implants are useful in patients with reduced bone height.

## Figures and Tables

**Figure 1:**
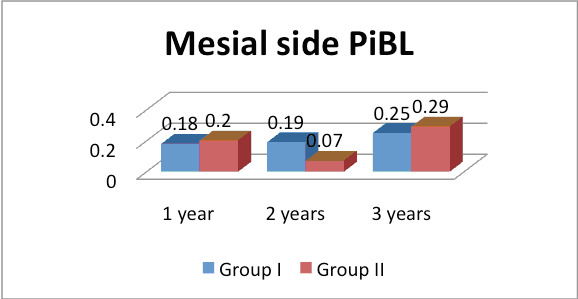
Assessment of peri-implant bone loss (PiBL) on mesial and distal side of implants in both groups

**Figure 2:**
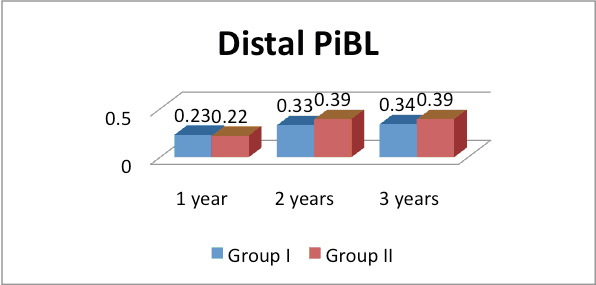
Assessment of peri implant bone loss (PiBL) on distal side of implants in both groups
